# Detection of Phocoena Pestivirus and Neutralizing Antibodies in Harbor Porpoises Found Dead at the North Sea and Baltic Sea Coasts

**DOI:** 10.1155/tbed/5525870

**Published:** 2026-07-23

**Authors:** Lars Söder, Denise Meyer, Nele Gremmel, Alexander Postel, Ursula Siebert, Luca Aroha Schick, Stephanie Groß, Jens Brackmann, Christine Baechlein, Benjamin Lamp, Till Rümenapf, Paul Becher

**Affiliations:** ^1^ Institute of Virology, University of Veterinary Medicine Hannover, Hannover, Germany, tiho-hannover.de; ^2^ Institute of Terrestrial and Aquatic Wildlife Research (ITAW), University of Veterinary Medicine Hannover, Hannover, Germany, tiho-hannover.de; ^3^ Department of Ecoscience, Aarhus University, Roskilde, Denmark, au.dk; ^4^ Food and Veterinary Institute Oldenburg, Lower Saxony State Office for Consumer Protection and Food Safety, Oldenburg, Germany; ^5^ Food and Veterinary Institute Braunschweig/Hannover, Lower Saxony State Office for Consumer Protection and Food Safety, Hannover, Germany; ^6^ Institute of Virology, Department of Veterinary Medicine, Justus-Liebig-University, Giessen, Germany, uni-giessen.de; ^7^ Institute of Virology, University of Veterinary Medicine Vienna, Vienna, Austria, vetmeduni.ac.at

**Keywords:** genome prevalence, organ homogenate, organ liquid, pestivirus, Phocoena pestivirus, virus neutralization test

## Abstract

In 2019, a marine pestivirus, Phocoena pestivirus (PhoPeV), family *Pestiviridae*, was described for the first time. To date, the impact of PhoPeV infections on harbor porpoises is still unknown. Therefore, a screening study was carried out on harbor porpoises found dead on the German North Sea and Baltic Sea coasts during 2021–2025. Analysis of organ samples collected from 84 harbor porpoises by reverse transcription (RT)‐PCR showed the presence of PhoPeV RNA in samples from five animals (5.9%). Infectious virus was isolated from two of these samples. Phylogenetic analysis showed a close relationship to previously reported PhoPeV isolates. Moreover, a virus neutralization assay has been established using organ liquid and organ homogenate matrices instead of the standard serum sample matrix. Samples from seven out of 72 animals tested positive for neutralizing antibodies against PhoPeV including four samples with an antibody titer ≥ 320 ND_50_. Cross‐neutralization studies including classical swine fever virus (CSFV), border disease virus (BDV), as well as Bungowannah pestivirus (BuPV), and Linda virus (LindaV), both viruses being most closely related to PhoPeV, showed no significant serological relatedness to PhoPeV. In conclusion, both PhoPeV RNA and neutralizing antibodies against PhoPeV have been detected in animals found dead in the North Sea and Baltic Sea over the last 5 years, indicating a continuous circulation of PhoPeV in the harbor porpoise population for at least several years.

## 1. Introduction

The genus *Orthopestivirus* (family *Pestiviridae*) includes causative agents of economically important animal diseases such as classical swine fever virus (CSFV, species *Orthopestivirus suis*), bovine viral diarrhea virus‐1 (BVDV‐1, species *Orthopestivirus bovis*), bovine viral diarrhea virus‐2 (BVDV‐2, species *Orthopestivirus tauri*), and border disease virus (BDV, species *Orthopestivirus ovis*), as well as a growing number of additional pestivirus species detected in various domestic and wild animal hosts [1–5]. In 2019, the first marine pestivirus, Phocoena pestivirus (PhoPeV, species *Orthopestivirus phocoenae*), was described in harbor porpoises, *Phocoena phocoena* (Linnaeus, 1758) of the Dutch North Sea and later in animals of the Baltic Sea region with genome detection rates of 9% and 1.3%, respectively [6, 7]. The impact of PhoPeV on the health of harbor porpoises and its host range remain unknown [6–8]. For pestiviruses of ruminant and porcine host species like BVDV and CSFV, it is known that infected animals shed the virus in all body secretions and feces [9, 10]. Pestiviruses can be transmitted either by direct contact with infected animals or by indirect contact through contaminated food or surfaces, resulting in acute infections. As described for CSFV and BVDV, horizontal infections can lead to a variety of clinical signs of acute disease, immunosuppression, or inapparent infections. In addition, infection of pregnant animals by pestiviruses can result in vertical transmission and intrauterine infection of the fetus [11]. Depending on the time of gestation, intrauterine infections can cause abortion, other reproductive disorders, and the establishment of persistent infections in the offspring due to a specifically acquired immunotolerance of the fetus against the infecting pestivirus [3, 11, 12]. These persistently infected (PI) animals shed continuously large amounts of the pathogen throughout their lifespan, leading to a highly contaminated environment and constant intraherd virus circulation [2, 11–14]. It is still unknown whether intrauterine infections also play an important role in the spread and circulation of PhoPeV in the harbor porpoise population.

Pestiviruses possess a single‐stranded positive‐sense RNA genome of ~12 kb organized as one large open reading frame (ORF) flanked by untranslated regions (UTRs). The viral polyprotein is processed into 12 mature proteins (N^pro^, C, E^rns^, E1, E2, p7, NS2, NS3, NS4A, NS4B, NS5A, and NS5B) [2]. Compared to other families of the order *Amarillovirales*, pestiviruses express two unique viral proteins, the N‐terminal autoprotease (N^pro^) and the glycoprotein E^rns^ (envelope glycoprotein ribonuclease‐secreted). Both viral proteins interact with the host innate immune response, thereby overcoming the antiviral defense. Notably, PhoPeV lacks the N^pro^ coding region, resulting in the expression of a shorter pestiviral polyprotein [6, 7]. N^pro^ directly affects host immunity by mediating proteasomal degradation of interferon‐regulatory transcription factor 3 (IRF3) and through interference with IRF7 in plasmacytoid dendritic cells (pDCs) [15–17]. In contrast to other noncytopathic pestiviruses, the lack of N^pro^ expression in cells infected with PhoPeV results in the absence of IRF3 degradation and in the expression of interferon‐stimulated genes (ISGs), such as Mx1 [8].

According to phylogenetic analyses, PhoPeV is most closely related to Bungowannah pestivirus (BuPV, species *Orthopestivirus australiaense*) and Linda virus (LindaV, species *Orthopestivirus steiermarkense*) [5, 7], which have caused local outbreaks on pig farms in Australia and Austria, respectively [18, 19]. While BuPV exhibits a broad host cell tropism, PhoPeV infects porcine kidney cells (porcine kidney‐15 [PK‐15]) and swine kidney 6 (SK6), Madin‐Darby bovine kidney (MDBK) cells, sheep fetal thymus (SFTR) cells, as well as Crandel‐Rees feline kidney (CRFK) cells as described for CSFV [8]. After infection with pestiviruses, the animals produce neutralizing antibodies against envelope glycoprotein E2 and, to a minor extent, also against E^rns^ [20–22]. Previous studies have shown that these antibodies are not only specific for the inoculum virus but can also cross‐neutralize other pestiviruses [23–25].

To date, the prevalence of PhoPeV in the harbor porpoise population of the North Sea and Baltic Sea has been investigated in two studies that focused exclusively on the detection of PhoPeV genomes [6, 7]. In the present study, organ samples from various cetacean species found dead on the German North Sea and Baltic Sea coasts over the past 5 years were analyzed. These samples were tested for the PhoPeV genome, and genome‐positive samples were used for the isolation of infectious virus. For the first time, a virus neutralization test (VNT) detecting neutralizing antibodies in organ matrices was established. To investigate the serological relatedness of PhoPeV to other pestiviruses, especially to the most closely related pestiviruses, BuPV and LindaV, cross‐neutralization was performed.

## 2. Materials and Methods

### 2.1. Samples

Screening for PhoPeV genomes was performed by analyzing organ samples from 84 harbor porpoises (*Phocoena phocoena*) provided by the Institute of Terrestrial and Aquatic Wildlife Research (ITAW; University of Veterinary Medicine Hannover, Germany) and the Lower Saxony State Office for Consumer Protection and Food Safety (LAVES, Oldenburg and Hannover, Germany). Of all the tested animals, 44 were found dead at the coast of the Baltic Sea, 30 at the coast of the North Sea, and 10 at the Elbe estuary close to the North Sea. Samples that were provided by ITAW were collected within the framework of the marine mammal stranding network of the county of Schleswig‐Holstein, Germany [26]. The animals were transported to the ITAW (University of Veterinary Medicine Hannover, Buesum, Germany) or to the Food and Veterinary Institute (LAVES, Oldenburg, Germany) for detailed necropsy. The collected samples were stored at −70°C until further analysis. In the present study, the sample matrices of lung and brain were examined.

### 2.2. Porcine Cell Lines and Pestivirus Strains

For the isolation of infectious PhoPeV and for the detection of neutralizing antibodies against PhoPeV and LindaV, SK6 cells were used. VNTs with CSFV, BuPV, and BDV were carried out on the PK‐15 cell line. Both cell lines were cultured in minimum essential medium (MEM; Gibco Thermo Fisher, Waltham, MA, USA) containing 1% penicillin/streptomycin and supplemented with 5% or 10% fetal bovine serum (FBS). Only FBS batches that tested negative for pestivirus genome contamination by reverse transcription (RT)‐PCR and did not contain neutralizing antibodies against BVDV were utilized.

For the analysis of serological relatedness to other pestiviruses, cross‐neutralization assays were performed using the following pestivirus strains: PhoPeV strain 43720 from the Baltic Sea [6, 8], BuPV (kindly provided by P. Kirkland; Elizabeth Macarthur Agriculture Institute, Menangle, New South Wales, Australia), LindaV strain Austria1, BDV‐1 strain Frijters (kindly provided by A. J. de Smit; Institute for Animal Science and Health, Lelystad, The Netherlands), as well as the CSFV strains Alfort/187 (CSF0902) and Diepholz (CSF0104). These CSFV strains were obtained from the CSFV collection of the EU and WOAH Reference Laboratory for CSF (Institute of Virology, University of Veterinary Medicine, Hannover, Germany).

### 2.3. RNA Preparation and Real‐Time RT‐PCR

To investigate the distribution of PhoPeV, 84 harbor porpoises were tested for PhoPeV RNA. For this purpose, the corresponding organ samples were homogenized in lysis buffer RA1 (Macherey‐Nagel GmbH & Co. KG, Düren, Germany) supplemented with 1% β‐mercaptoethanol using the Fisherbrand Bead Mill 24 (Thermo Fisher) with the following setup: 6.0 m/s, max. 45 s, one cycle. Afterwards, RNA isolation was performed automatically applying the IndiMag Pathogen Kit (Indical Bioscience, Leipzig, Germany) or manually using the NucleoSpin RNA kit (Macherey‐Nagel GmbH & Co. KG) according to the manufacturer’s descriptions. The detection of PhoPeV RNA followed the protocol that has been previously described [6]. Initially, RNA was prepared from organ pools (lung and brain) of each animal. If this pool tested positive for PhoPeV RNA, the RNA was isolated from each organ separately and tested by real‐time RT‐PCR.

### 2.4. Nucleotide Sequencing and Phylogenetic Analysis

**Figure 1:**
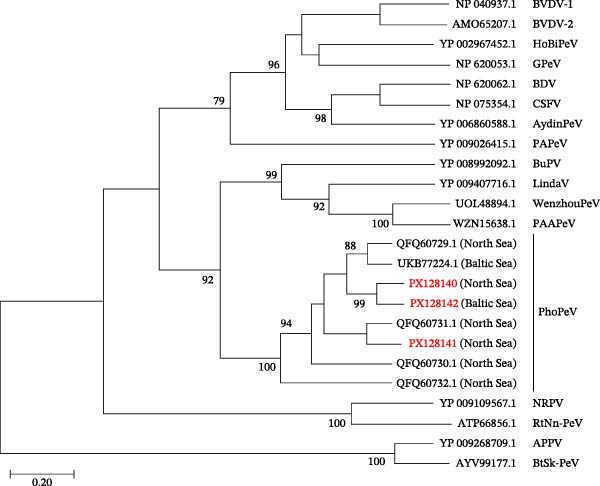
Phylogenetic analysis of pestivirus E2 protein sequences. Maximum‐likelihood phylogenetic tree based on complete E2 sequences of Phocoena pestivirus (PhoPeV) and other representative members of the family *Pestiviridae* was generated as described in Section 2. The sequences of the PhoPeV isolates that were newly generated in this study are highlighted in red (PX128140, PX128141, PX128142). For PhoPeV sequences, geographic origin (North Sea, Baltic Sea) is included in parentheses. Bootstrap values ≥ 70 are indicated. Numbers at the end of each branch indicate the GenBank accession numbers of the individual sequences. APPV, atypical porcine pestivirus, AydinPeV, Aydin pestivirus, BVDV‐1, bovine viral diarrhea virus‐1, BVDV‐2, bovine viral diarrhea virus‐2, BDV, border disease virus, BtSk‐PeV, bat pestivirus, BuPV, Bungowannah pestivirus, CSFV, classical swine fever virus, GPeV, pestivirus giraffe‐1, HoBi PeV, HoBi pestivirus, LindaV, Linda virus, NRPV, Norway rat pestivirus, PAAPeV, porcine abortion‐associated pestivirus, PAPeV, pronghorn antelope pestivirus, RtNn‐PeV, rodent pestivirus, WenzhouPeV, Wenzhou Pipistrellus abramus pestivirus 1.

Complementary DNA (cDNA) was synthesized from the samples that tested positive for PhoPeV RNA using the ThermoScientific RevertAid RT Kit (Thermo Fisher). Two conventional PCRs were conducted, one targeting the N^pro^ and the other one targeting the E2 coding region. To confirm the lack of the N^pro^ coding region, primers that bind to the 5′‐UTR and in the capsid protein coding region were used [7]. For phylogenetic analysis, the nucleotide sequence of the complete E2 coding region was amplified using two primer pairs (PhoPeV_E2_1 fwd: 5′‐CAGTTGCTTTTCTGCCGCT‐3′ and PhoPeV_E2_1 rev: 5′‐GCACACATTATTTCTGCAACAGG‐3′; PhoPeV_E2_2 fwd: 5′‐CACATTGGAGCTTACCACTAT‐3′ and PhoPeV_E2_2 rev: 5′‐AACAACCCTAGTATCCTTCTC‐3′) and the HighQ ALLin Mega HiFi Red Mastermix (HighQu, Kraichtal, Germany). PCR products were purified (ThermoScientific GeneJET Gel Extraction Kit; Thermo Fisher) and analyzed by Sanger sequencing (LGC Genomics GmbH, Berlin, Germany). The sequences were evaluated by software GENtle, Version 1.9.4.0 [27]. The E2 coding sequences were translated into amino acid (aa) sequences, and phylogenetic analyses were performed using the complete E2 protein sequences of 16 representative pestivirus strains: BVDV‐1 NADL, BVDV‐2 91 W, HoBi pestivirus, BDV X818, CSFV Eystrup, atypical porcine pestivirus (APPV) Bavaria S5/9, Norway rat pestivirus (NRPV) NYC‐D23, Aydin pestivirus Aydin/04‐TR, pestivirus giraffe‐1 H138, pronghorn antelope pestivirus, BuPV, LindaV, bat pestivirus BtSk‐PestV‐1/GX2017, rodent pestivirus (RtNn‐PestV) HuB2014, Wenzhou Pipistrellus abramus pestivirus 1, and porcine abortion‐associated pestivirus (PAAPeV) together with five previously reported PhoPeV isolates. The respective GenBank accession numbers are provided in Figure 1. Evaluation and visualization of phylogenetic analysis (Figure 1) were performed using the Maximum likelihood method and Jones‐Taylor‐Thornton (JTT) matrix‐based model with 1000 bootstrap replications using Molecular Evolutionary Genetics Analysis Version 12 (MEGA12) [28].

### 2.5. Virus Isolation

For homogenization, a pea‐sized amount of lung and brain samples from each animal (10–30 mg) was added to separate tubes containing ceramic beads and 1.2 mL of cell culture medium with 10% penicillin/streptomycin. Homogenization was performed using the Fisherbrand Bead Mill 24 (Thermo Fisher), and the setup used for RNA preparation was applied. The organ homogenates were incubated at room temperature for 1 h and centrifuged for 1 min at 4°C at 13,000 rounds per minute (rpm). Supernatants (sample dilutions: undiluted, 1:5 up to 1:100) were inoculated on SK6 cells [29]. Two serial passages, 72 h of incubation each, were performed. After the second passage, the presence of viral antigen was assessed by indirect immunoperoxidase staining using monoclonal antibody (MAb) WB166 [30].

### 2.6. VNT and Cross‐Neutralization Test

**Figure 2:**
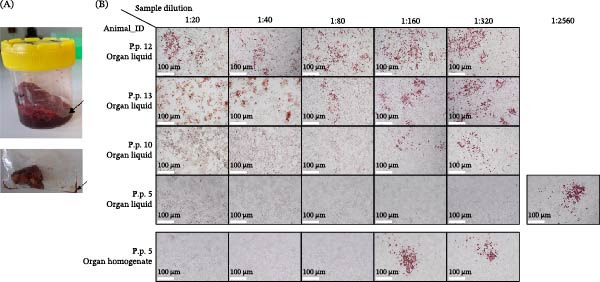
Establishment of a virus neutralization test (VNT) for the detection of neutralizing antibodies against PhoPeV. VNT was performed for 72 animal samples using the porcine cell line SK6 and the PhoPeV Baltic strain 43720 [6]. (A) Sample tubes with organ liquid (marked by arrows). (B) The sample dilution series of organ liquids started with 1:20 due to cytotoxic effects in the dilutions 1:5 and 1:10. In exceptional cases, unspecific cytotoxic effects on the cell layer were observed up to sample dilution 1:40 (e.g., antibody‐negative sample P.p. 13 and antibody‐positive sample P.p. 10). In addition, organ homogenates were prepared in cell culture medium containing antibiotics without FBS. The initial sample dilution is 1:5 (e.g., sample P.p. 5). VNTs were evaluated 72 h postinfection by peroxidase‐linked antibody staining using monoclonal antibody WB166 [30]. P.p., *Phocoena phocoena*.

The VNT represents the gold standard for the detection of antibodies against pestiviruses. In general, serum represents the preferred sample matrix for the detection of antibodies. However, this sample matrix can only be obtained in exceptional cases from animals that were found dead. Therefore, a VNT was established using organ liquid or organ homogenates (Figure 2A). In total, organ samples from 72 out of 84 harbor porpoises were available for analysis by VNT. The organ homogenates were prepared from lung (*n* = 67) or brain (*n* = 5) tissues using 0.5 mL of cell culture medium containing 10% penicillin/streptomycin. In addition, for 23 out of these 72 organ samples, it was possible to collect organ liquid directly from the tubes filled with the organ material (Figure 2A). The initial sample dilution in the VNT of the organ homogenates was 1:5. When testing organ liquids, the use of initial sample dilutions of 1:5 and 1:10 frequently resulted in cytotoxic effects. Therefore, the organ liquids (*n* = 23) were analyzed in the present VNT, starting with a 1:20 dilution. The VNT was performed according to the EU and WOAH Manual of Diagnostic Tests for the Detection of CSF [29] using the previously described PhoPeV Baltic strain 43720 [6, 8]. Neutralizing antibody titers were determined using the Spearman‐Kaerber method [31, 32] and were expressed as initial sample dilution. The data were illustrated using GraphPad Prism Version 10.4.1 (GraphPad Software Inc., Boston, MA, USA).

In addition, cross‐neutralization tests were performed, which have been widely used to show similarities and differences between pestiviruses and represent one important criterion for pestivirus classification [1, 23]. The organ liquid of the harbor porpoise, P.p. 5 (initial sample dilution 1:20) was analyzed together with porcine sera (initial serum dilution 1:5) that were collected from pigs experimentally infected with BuPV, LindaV, BDV strain Frijters, or CSFV strains Alfort/187 and Diepholz, respectively. These porcine sera were obtained from the serum collection of the EU and WOAH Reference Laboratory for CSF, with the exception of the LindaV‐specific antisera, which have been described previously [33]. The VNTs were performed using the homologous pestivirus strains, including the PhoPeV strain 43720 [6], CSFV Alfort/187, CSFV Diepholz, and BDV Frijters strains, as well as BuPV and LindaV, which are genetically more closely related to PhoPeV than to other pestiviruses.

### 2.7. Indirect Immunoperoxidase and Immunofluorescence (IF) Staining

Viral antigen was detected by peroxidase‐linked antibody assay (PLA) or IF staining (detection of BuPV antigen), as described previously [8, 29]. For the detection of the PhoPeV antigen, the MAb WB166 [30] was applied in a 1:500 dilution. The BuPV antigen was detected using a porcine polyclonal BuPV‐specific antiserum (diluted 1:12,000). MAb 11D5 (diluted 1:7.5) was used for the detection of the LindaV antigen [19]. Infections of cells with CSFV strain Alfort/187, CSFV strain Diepholz, and BDV‐1 strain Frijters were analyzed using the MAb BVD/C16 (diluted 1:50; [34]). Conjugated polyclonal rabbit anti‐mouse immunoglobulins/HRP (Dako A/S, Glostrup, Denmark) and Cy3‐affiniPure goat anti‐pig IgG (H + L) (Dianova, Hamburg, Germany) were diluted 1:250, respectively. Evaluation of viral growth was performed by bright‐field or IF microscopy using the Olympus inverse microscope CKX53 (Olympus K.K., Tokyo, Japan) and Nikon Eclipse Ti (Nikon Corporation, Minato, Japan). Images were collected using the cellSens imaging software (cellSens Version 4.3, Olympus K.K.) and NIS‐Elements AR software (NIS‐Elements AR Version 5.21.03, Nikon Corporation).

## 3. Results

### 3.1. Detection of PhoPeV in Harbor Porpoises, Virus Isolation, and Phylogenetic Analysis

Two animals from the North Sea (one female and one male) and three female animals from the Baltic Sea region tested positive for PhoPeV RNA, which corresponds to a detection rate of PhoPeV RNA of 5.9% (Table 1). An infectious virus was obtained from two out of the five PCR‐positive samples (P.p. 2 and P.p. 4), which showed a Cq value of <27. These samples were collected from harbor porpoises found dead on the North Sea coast (Table 1). No infectious virus was isolated from samples with Cq values of ≥36 (P.p. 1, P.p. 3, P.p. 5). RT‐qPCR‐negative samples were not subjected to virus isolation. As described for other PhoPeV isolates, the genomes of both new PhoPeV isolates do not encode the entire N^pro^. For these virus isolates and for an additional sample, which tested positive for PhoPeV RNA (P.p. 5), complete E2 coding sequences were generated (GenBank Accession Numbers: PX128140‐42). Attempts to amplify the complete E2 encoding region from the remaining two PhoPeV RNA‐positive samples failed (Table 1). Phylogenetic analysis based on complete E2 protein sequences showed that the PhoPeV isolates described here are closely related to the previously reported PhoPeV strains. The PhoPeV isolate of the year 2023 (PX128141) clustered together with the virus isolates previously detected in animals from the North Sea (Figure 1).

**Table 1:** Overview of the harbor porpoises tested for PhoPeV RNA and/or for neutralizing antibodies against PhoPeV.

Animal ID	Year	Origin	Animal gender	RT‐qPCR (Cq value)		E2 sequence	Accession number	Virus isolation	Virus neutralization test (ND_50_)	
				Lung	Brain				Organ liquid	Organ homogenate
P.p. 1	2023	Baltic Sea	Female	36	36	−	—	Negative	<20	<5
P.p. 2	2022	North Sea	Male	23	23	+	PX128140	Positive	<20	<5
P.p. 3	2022	Baltic Sea	Female	36	No Cq	−	—	Negative	<20	<5
P.p. 4	2023	North Sea	Female	26	27	+	PX128141	Positive	<20	<5
P.p. 5	2023	Baltic Sea	Female	39	38	+	PX128142	Negative	1920	320
P.p. 6	2022	Baltic Sea	Female	No Cq	No Cq	−	—	n.t.	640	60
P.p. 7	2023	Baltic Sea	Female	No Cq	No Cq	−	—	n.t.	410	60
P.p. 8	2022	North Sea	Female	No Cq	No Cq	−	—	n.t.	410	60
P.p. 9	2022	Baltic Sea	Female	No Cq	No Cq	−	—	n.t.	80	40
P.p. 10	2023	Baltic Sea	Female	No Cq	No Cq	−	—	n.t.	80	15
P.p. 11	2024	Baltic Sea	Female	No Cq	No Cq	−	—	n.t.	n.a.	20

*Note:* + = complete E2 sequence determined; − = complete E2 sequence not determined/not applicable.

Abbreviations: n.a., not available; n.t., not tested; P.p., *Phocoena phocoena*.

### 3.2. Detection of Neutralizing Antibodies Against PhoPeV

A VNT was established using organ liquids or organ homogenates. In total, organ samples from 72 out of 84 harbor porpoises were tested for neutralizing antibodies against PhoPeV by VNT. When testing organ liquids (*n* = 23), the samples were tested in a 1:20 dilution to avoid cytotoxic effects in the cell culture. However, in exceptional cases, samples of poor quality can affect the cell layer up to a 1:40 dilution, as shown for sample P.p. 13 and P.p. 10. For sample P.p. 13, destroyed cell morphology was observed, and red‐stained cell debris was detected in sample dilutions of 1:20 and 1:40 (Figure 2B). This stained cell debris can suggest a staining of the pestiviral antigen in the cytoplasm and consequently would argue against the presence of neutralizing antibodies in this sample.

**Figure 3:**
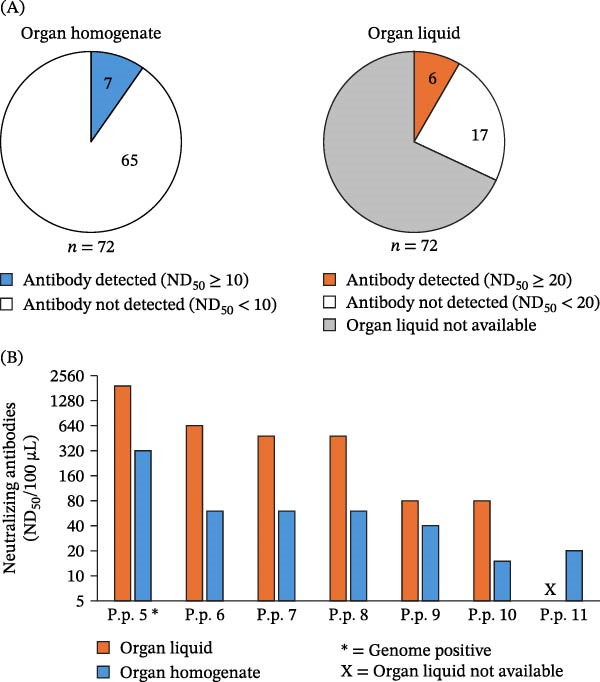
Detection of neutralizing antibodies against PhoPeV using the virus neutralization test (VNT). (A) Depending on organ availability, lung (*n* = 67) and brain (*n* = 5) tissue homogenates from harbor porpoises (*n* = 72) were analyzed in the VNT. In addition, organ liquid was collected from 23 out of these 72 organ samples and applied in the VNT. (B) Neutralizing antibody titers detected in organ homogenates (blue) and organ liquids (red) of seven animals. Illustration was performed using GraphPad Prism software Version 10.4.1. P.p., *Phocoena phocoena*.

In total, antibody titers between 80 and 1920 ND_50_/100 µL were detected in six out of 23 organ liquid samples collected from harbor porpoises (Figure 3A), with one sample also being tested positive for the PhoPeV genome (P.p. 5, Table 1). For samples containing no neutralizing antibodies against PhoPeV, viral antigen was detected in the VNT from the first sample dilution onwards (e.g., sample P.p. 12). To exclude the possibility that virus growth was inhibited independently of the presence of neutralizing antibodies, organ liquid of sample P.p. 5 with a high antibody titer was tested in a VNT using the pestivirus strain CSFV Alfort/187. No inhibition of virus growth was detected. The VNT with CSFV Alfort/187 showed that viral antigen was already detected after incubation with an initial sample dilution of 1:20 (data not shown). The results thus show that PhoPeV is neutralized by antibodies present in organ liquids and that the specific neutralizing effect is absent after a certain dilution of the sample matrix (Figure 2B).

In addition, organ homogenates from the tissue samples of 72 animals were analyzed. For evaluation, organ homogenates were tested in comparison to organ liquids of the six animals tested positive for PhoPeV antibodies (ND_50_ = 80–1920). This verification study showed that the neutralizing antibody titers in organ homogenates were up to tenfold lower than the titers in organ liquids (Figure 3B). This difference can be explained by a dilution effect of the antibody‐containing matrix as the organ homogenates were prepared by adding a substantial amount (500 µL) of cell culture medium. However, due to this dilution effect and the application of antibiotics in the cell culture medium, no or only minimal cytotoxic effects were observed in cell cultures inoculated with a sample dilution of 1:5 (Figure 2B). All organ liquid samples that tested negative for neutralizing antibodies against PhoPeV (*n* = 17) were also negative in the VNT using the corresponding organ homogenates. For 49 out of the 72 animals, only organ homogenates could be investigated. One out of these 49 organ homogenates tested positive for neutralizing antibodies with a titer of 20 ND_50_/100 µL (Figure 3B). For the other 48 organ homogenates, neutralizing antibodies were not detectable (Figure 3A).

### 3.3. Antigenic Relatedness of PhoPeV to Other Pestiviruses

To evaluate the antigenic relatedness of PhoPeV to other pestiviruses, a cross‐neutralization test was performed including the PhoPeV strain 43720 [6], CSFV‐, and BDV strains as well as BuPV and LindaV. Cross‐neutralization was not observed for the CSFV strains Alfort/187 (genotype 1.1) and Diepholz (genotype 2.3), nor for BDV strain Frijters or BuPV. The porcine serum sample, which contains antibodies against LindaV, was tested negative in the PhoPeV VNT, as well as in the VNTs using the CSFV strains Alfort/187 and Diepholz and BuPV and BDV strain Frijters. Interestingly, the organ liquid collected from animal P.p. 5 (antibody titer against PhoPeV = 1920 ND_50_/100 µL) showed an antibody titer of 120 ND_50_/100 µL in the VNT using LindaV. The other organ liquid samples that were tested positive in the PhoPeV VNT (ND_50_ = 80–640; Table 1) showed no cross‐neutralization in any applied VNT.

## 4. Discussion

Initial characterization of PhoPeV showed that this virus replicates in the cell lines SK6, PK‐15, SFTR, and MDBK [8]. Porcine, bovine, and ovine cell lines are generally applied in pestivirus research and in cell culture‐based diagnostic methods [29]. To date, no serological screening studies have been conducted to detect antibodies against PhoPeV in harbor porpoises. One of the well‐established methods for the detection of virus‐specific antibodies is the VNT. Since PhoPeV replicates very efficiently in the porcine cell line SK6 [8], these cells were used to establish a PhoPeV VNT. The samples that were tested for the presence of neutralizing antibodies against PhoPeV were initially screened for PhoPeV RNA to confirm that the virus was still present in the harbor porpoise population of the German North Sea and Baltic Sea between 2021 and 2025. In total, 84 harbor porpoises were included in the present study. Five out of 84 samples tested positive for PhoPeV RNA, resulting in a genome detection rate of 5.9%, which corresponds to the previously reported prevalence in harbor porpoises of 9% (North Sea region) and 1.3% (Baltic Sea region) [6, 7]. These results indicate a consistent circulation of PhoPeV in the harbor porpoise population. Two new PhoPeV strains were isolated (Table 1). Analysis of the complete PhoPeV E2 sequences established from samples of three animals revealed a high similarity with the previously published PhoPeV sequences. These results indicate a relatively constant presence of the virus within the harbor porpoise population. In addition, a small number of samples from other cetacean species (one dolphin *Tursiops truncatus* [Montagu, 1821]; one dolphin *Delphinus delphis* Linnaeus, 1758; two minke whales *Balaenoptera acutorostrata* Lacépède, 1804; one sperm whale *Physeter macrocephalus* Linnaeus, 1758; and one humpback whale *Megaptera novaeangliae* [Borowski, 1781]) were tested for the PhoPeV genome (data not shown). As previously shown for various seal species, PhoPeV RNA could not be detected in these samples [6]. To improve our knowledge about the natural host range of PhoPeV and related viruses among marine mammals, a larger number of samples from cetaceans other than harbor porpoises and other marine mammal species remain to be investigated.

Serum is the preferred sample matrix regarding the detection of antibodies against viral pathogens. However, if this matrix is unavailable because the carcasses were found at a late stage after death, alternative sample matrices can be used instead. For example, antibodies against African swine fever virus were detected by ELISA using blood swabs, which were collected from wild boar carcasses [35]. In a similar approach, sample matrices, which contain blood, were used in the present study for antibody detection as an alternative to serum. The quality and quantity of most of the samples collected from the dead harbor porpoises found on the coast were poor and limited. Therefore, only samples from 72 out of 84 animals were investigated regarding the presence of neutralizing antibodies. Organ homogenates prepared from samples of 72 animals were analyzed in a PhoPeV VNT. In addition, organ liquids from 23 out of these 72 samples could be collected directly from the tubes in which the organ samples (lung) were stored. Six organ liquid samples and the corresponding six organ homogenates, as well as one additional organ homogenate (from a sample for which no organ liquid was obtained), tested positive for neutralizing antibodies against PhoPeV with titers ≥ 10 ND_50_/100 µL (Figure 3A). Four out of these seven samples showed an antibody titer ≥ 320 ND_50_/100 µL (Figure 3B). These results confirm that both sample matrices can be used as an alternative to serum. However, the application of organ homogenates in comparison to the organ liquid is less sensitive (Figure 3B). One possible reason for this observation is the dilution effect of the cell culture medium added to the organ sample prior to homogenization. Furthermore, the use of organ homogenates could be improved by using samples from organs with a higher blood content (e.g., spleen). In general, a serum sample is classified positive for neutralizing antibodies against a specific pestivirus if the antibody titer in the VNT is ≥10 ND_50_. This cut‐off value cannot be applied for the organ liquid samples used in the present PhoPeV VNT because the initial sample dilution was set to 1:20 due to the detected cytotoxic effects. Therefore, the cut‐off value for this sample category was set to ≥20 ND_50_. Moreover, in rare cases, cytotoxic effects were observed in sample dilutions higher than 1:20. For the antibody‐negative sample P.p. 13, cytotoxic effects were detected up to a sample dilution of 1:40, and the stained cell debris may suggest a specific detection of PhoPeV antigen (Figure 2B). In contrast, samples that contained neutralizing antibodies and induced cytotoxic effects did not show any staining in the cytoplasm or in the cell debris. In conclusion, a reliable evaluation of antibody‐positive samples was only possible from the dilution onwards at which an intact cell layer was present, allowing the assessment of the staining of single cells. All antibody‐positive samples showed an intact cell layer in the previous sample dilution before virus antigen was detected in the subsequent dilution.

With one exception, all animals that tested positive for neutralizing antibodies tested negative for PhoPeV RNA. As shown for CSFV and BuPV, detection of neutralizing antibodies might suggest that these animals were infected with PhoPeV and subsequently developed a protective humoral immune response, leading to viral clearance [3, 36, 37]. One animal (P.p. 5) tested positive for both PhoPeV RNA and neutralizing antibodies, which might be indicative of an acute infection at a stage where an immune response was already developed but viral RNA genomes were still detectable [36, 37]. No neutralizing antibodies were detected in the remaining four samples that tested positive for PhoPeV RNA. An infectious virus was isolated from two of these PhoPeV RNA‐positive samples. It can be speculated that these animals were sampled during an early stage of acute infection with PhoPeV [36]. Alternatively, the PhoPeV RNA‐positive samples might have been obtained from PI animals. It is well described that infection of pregnant animals with noncytopathogenic pestiviruses can result in intrauterine infection of the fetus by vertical transmission [11]. In early stages of gestation, intrauterine infections in cattle and other ungulates can result in specific immunotolerance against the infecting virus and persistent infections of the offspring. These PI animals continuously shed large amounts of infectious virus, remain negative for antibodies against the corresponding pestivirus, and can develop a humoral immune response only after an additional infection with an antigenically different pestivirus. The present data do not allow a differentiation of an acute or persistent infection of the harbor porpoises with PhoPeV. However, the high number of PhoPeV genome‐positive animals observed over several years may suggest the occurrence of persistent infections in the harbor porpoise population.

Several studies have described antigenic cross‐reactivity between different pestivirus species, for example, between CSFV and BDV, BVDV‐1, or BVDV‐2 [23–25, 38–41]. This highlights the antigenic relatedness between these pestiviruses. As shown in the phylogenetic tree, which is based on the immunodominant protein E2, PhoPeV is more closely related to BuPV and LindaV than to CSFV, BDV, BVDV‐1, and BVDV‐2 (Figure 1). As expected, no cross‐neutralization was detected between PhoPeV and the genetically distantly related pestivirus strains CSFV Alfort/187, CSFV Diepholz, and BDV Frijters and *vice versa*. In addition, no cross‐neutralization was detected with BuPV. Interestingly, the organ liquid with the highest titer of PhoPeV‐specific neutralizing antibodies (1920 ND_50_/100 µL) was also tested positive in the LindaV‐specific VNT (120 ND_50_/100 µL). The aa sequence identity of PhoPeV‐ and LindaV E2 is ~49% depending on the virus isolate. However, the serum sample containing a high titer of antibodies against LindaV showed no cross‐neutralization against PhoPeV. In general, cross‐neutralization tests are performed using exactly the virus isolates which induced the production of neutralizing antibodies after infection. Due to the lack of a pair of PhoPeV‐positive and PhoPeV antibody‐positive samples from the same animal, the PhoPeV isolate used for VNT is not completely homologous to the antibodies detected in the organ fluid sample.

## 5. Conclusions

In conclusion, the results of the present study provide further insights into the distribution of PhoPeV, suggesting consistent circulation in harbor porpoise populations in the North Sea and Baltic Sea regions. Furthermore, the development of a PhoPeV‐specific VNT using alternative sample matrices provides additional insights into the distribution of PhoPeV, as well as an initial understanding of the virus–host interaction regarding humoral immune responses. Additional screening of different cetacean species and habitats as well as the establishment of harbor porpoise cell lines will extend the knowledge of the impact of PhoPeV on the harbor porpoise population.

## Author Contributions

Conceptualization, supervision: Denise Meyer and Paul Becher. Data curation, visualization, writing – original draft: Lars Söder and Denise Meyer. Formal analysis, methodology: Lars Söder, Denise Meyer, and Paul Becher. Funding acquisition, project administration: Paul Becher. Resources: Nele Gremmel, Alexander Postel, Ursula Siebert, Luca Aroha Schick, Stephanie Groß, Jens Brackmann, Christine Baechlein, Benjamin Lamp, Till Rümenapf, Denise Meyer, and Paul Becher. Writing – review and editing: Nele Gremmel, Alexander Postel, Ursula Siebert, Luca Aroha Schick, Stephanie Groß, Jens Brackmann, Christine Baechlein, Benjamin Lamp, Till Rümenapf, and Paul Becher.

## Funding

This study was supported by the Deutsche Forschungsgemeinschaft (DFG, German Research Foundation) (Grant 398066876/GRK 2485/2). Open Access funding enabled and organized by Projekt DEAL.

## Disclosure

All authors have read and agreed to the published version of the manuscript.

## Ethics Statement

The collection of carcasses and initial sampling was done opportunistically and none of the animals were euthanized or killed for the study. No consent from an Animal Use Committee is required when dealing with dead animals, as was the case here. The serum sample, which is positive for antibodies against LindaV and was used for cross‐neutralization assays, was provided by the Institute of Virology, University of Veterinary Medicine Vienna, Austria. The corresponding animal experiment was approved by the ethics committee of the University of Veterinary Medicine, Vienna and the Federal Ministry of Science, Research and Economy according to the §§ 26 ff. of the Austrian Animal Experiments Act from 2012 (Permission Code BMWF‐68.205/0130‐WF/V/3b/2017, Permission Date: July 07, 2017). The remaining serum samples were obtained from the serum collection of the EU and WOAH Reference Laboratory for CSF and were collected from animal experiments that were made known to the Specialized Department of Animal Welfare Service of the Lower Saxony State Office for Consumer Protection and Food Safety (LAVES; Permit Numbers LAVES AZ 08A 538 and LAVES AZ 18/2761) according to the German Animal Welfare Act.

## Conflicts of Interest

The authors declare no conflicts of interest.

## Data Availability

The data that support the findings of this study are available from the corresponding author upon reasonable request.
